# All Light, Everywhere? Photoreceptors at Nonconventional Sites

**DOI:** 10.1152/physiol.00017.2023

**Published:** 2023-10-31

**Authors:** Audrey Mat, Hong Ha Vu, Eva Wolf, Kristin Tessmar-Raible

**Affiliations:** ^1^Max Perutz Labs, University of Vienna, Vienna BioCenter, Vienna, Austria; ^2^VIPS^2^, Vienna BioCenter, Vienna, Austria; ^3^Institute of Molecular Physiology, Johannes Gutenberg-University, Mainz, Germany; ^4^Institute of Molecular Biology, Mainz, Germany; ^5^Alfred Wegener Institute, Helmholtz Centre for Polar and Marine Research, Bremerhaven, Germany; ^6^Carl-von-Ossietzky University, Oldenburg, Germany

**Keywords:** chronobiology, cryptochrome, light, opsin, nonvisual photoreceptors

## Abstract

One of the biggest environmental alterations we have made to our species is the change in the exposure to light. During the day, we typically sit behind glass windows illuminated by artificial light that is >400 times dimmer and has a very different spectrum than natural daylight. On the opposite end are the nights that are now lit up by several orders of magnitude. This review aims to provide food for thought as to why this matters for humans and other animals. Evidence from behavioral neuroscience, physiology, chronobiology, and molecular biology is increasingly converging on the conclusions that the biological nonvisual functions of light and photosensory molecules are highly complex. The initial work of von Frisch on extraocular photoreceptors in fish, the identification of rhodopsins as the molecular light receptors in animal eyes and eye-like structures and cryptochromes as light sensors in nonmammalian chronobiology, still allowed for the impression that light reception would be a relatively restricted, localized sense in most animals. However, light-sensitive processes and/or sensory proteins have now been localized to many different cell types and tissues. It might be necessary to consider nonlight-responding cells as the exception, rather than the rule.

## Introduction: the Many Roles of Light and Photoreceptors

**FIGURE 1.**
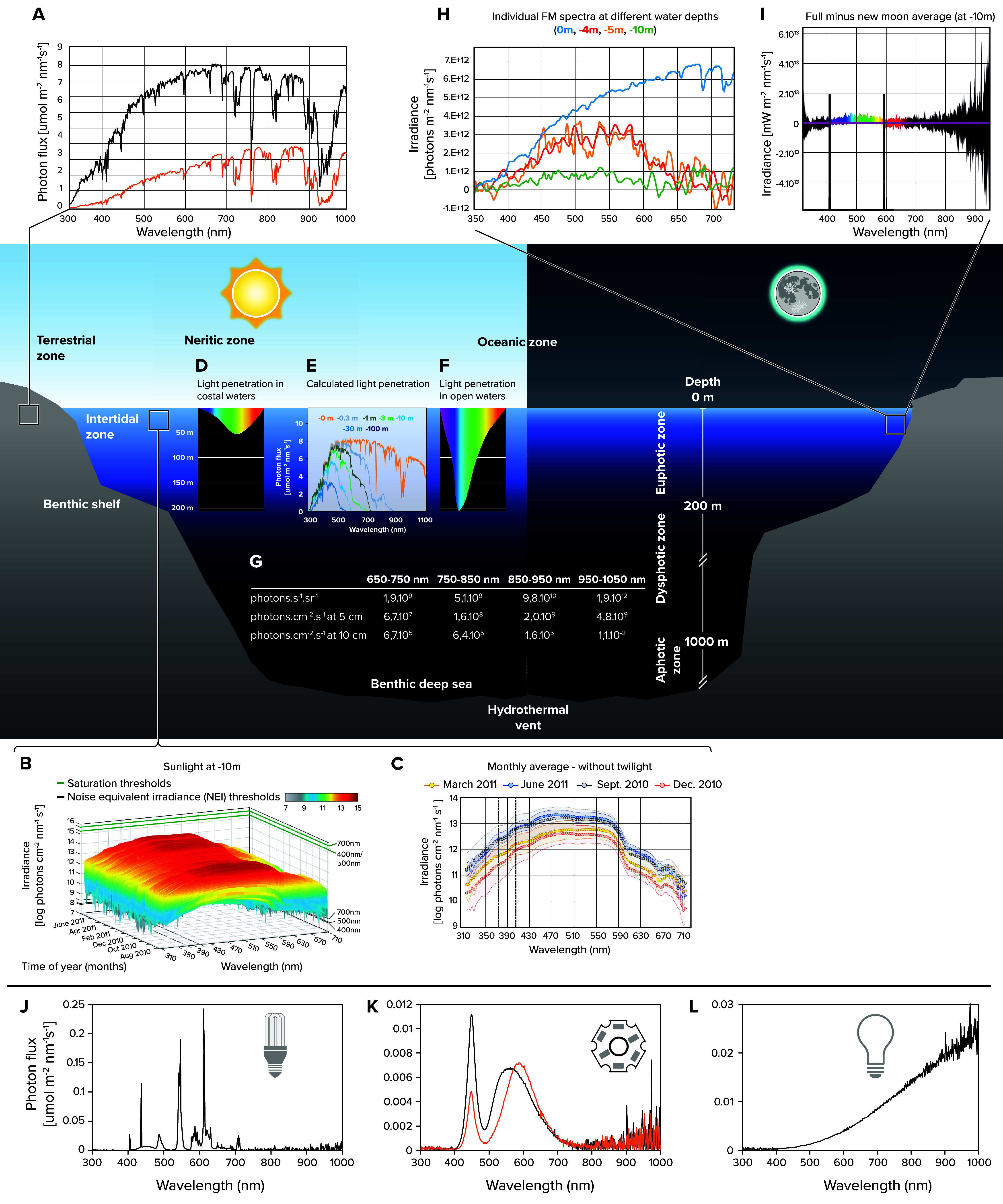
**Examples of natural light in different environments, as well as artificial light sources** *A*: representative spectrum and intensity for terrestrial light. Light blue: around noon; and orange: evening in Vienna, Austria. Image from Ref. 2 and used with permission from *PLoS One*. *B* and *C*: sunlight at 10-m depths in a coastal area without strong tidal changes (Ischia, Bay of Naples, Italy). Image from Ref. 3 and used with permission from *Nature Ecology & Evolution*. *B*: continuous measurement (every 15 min) over multiple months. *C*: example of seasonal differences in light spectra and intensity that can be used by organisms. *D*: average light penetration in coastal waters. Image from Ref. 4 and used with permission from Wiley-Blackwell. *E*: theoretically calculated light intensities at different water depths. Image from Ref. 5 and used with permission from the *International Journal of Astrobiology*. *F*: average light penetration in open oceans. Image from Ref. 4 and used with permission from Wiley-Blackwell. *G*: irradiance at the Hole-to-Hell hydrothermal vent on the East Pacific Rise. Image adapted from Ref. 6, with permission from *Geophysical Research Letters*. *H*: full moon (FM) spectra on land and at different water depths in a coastal environment. Images from Refs. 3 and 7 and used with permission from *Nature Ecology & Evolution* and *Proceedings of the National Academy of Sciences USA*, respectively. *I*: average moon spectrum (full moon values minus new moon values) at −10 m in a coastal environment at the Bay of Naples, Italy. Nocturnal data from Ref. 3. *J*–*L*: spectra of different artificial light sources: compact fluorescent lamp (CFL; *J*), LED (*K*), and incandescent (Tungsten) lamp (*L*). Image from Ref. 2 and used with permission from *PLoS One*.

For this review, we consider wavelength between 280 nm (conventional start of UVB) and 750 nm (red light), of which usually the range between 380 and 700 nm is considered to be in the human visible range, although even this range is debated (1). This wavelength selection also covers the range of currently known important nonvisual light sensory processes. For animals, light harbors several important pieces of information. It encodes several aspects of temporal information, i.e., time of day, lunar cycle, and season, but also acute weather conditions and for marine animals relative spectrum and intensity inform about water depth, distance to coast, turbulences, and phytoplankton blooms (FIGURE 1).

Cellular responses to light can be subdivided into two main categories (indicated here as “first” and “second”): first, sunlight directly triggers biochemical reactions in the mammalian skin that create or destroy metabolically relevant compounds, such as the synthesis of the vitamin/hormone D_3_ from 7-dehydrocholesterol by UVB light (8). Another more recently uncovered example is the generation of urocanic acid by a light-dependent degradation of histidine (9). Urocanic acid can cross the blood-brain barrier and neuronal membranes, impact glutamate levels in excitatory neurons, and ultimately lead to changes in learning and memory (9).

In neurons of *Drosophila melanogaster*, light can directly impact flavine adenine dinucleotide (FAD), an important cofactor for enzymes in cellular redox reactions, which is also a cofactor in photoreceptor proteins (see below). Moreover, high levels of free FAD are sufficient to mediate blue light-dependent magnetic sensitivity, which can but does not have to be potentiated by the COOH terminus of cryptochrome, a photoreceptor protein (10, 11).

Second, when talking about photoreceptors, this typically implies another path of light impact on biological systems: proteins optimized for photon capture by a (noncovalently or covalently) bound cofactor, such as retinal or FAD. In animals, there are so far only two protein groups known that can function as such photoreceptors, opsins (via retinal), and cryptochromes (via FAD). Of note, when studied in detail, other eukaryote groups exhibit a much wider variety of photoreceptors (12, 13). A typical categorization is by the chromophore cofactor and its photochemistry: flavin mononucleotide (FMN) embedded into a light-oxygen-voltage domain (LOV), FAD, and sometimes 5,10-methenyltetrahydrofolate (MTHF) provide light sensitivity to cryptochromes (cry), and FAD is also present in the blue light sensor using FAD (BLUF); photoactive yellow protein (PYP) uses a p-coumaric acid (pCA) chromophore; opsins bind retinal; and the phytochrome (phy) class uses a tetrapyrrole as the chromophore and CarH uses cobalamin (vitamin B12) (12–14). However, this categorization can be confusing as protein members within one group are not necessarily evolutionarily related, as exemplified for fungal versus animal rhodopsins. Both groups of photoreceptors use retinal as cofactor and are transmembrane proteins, but their signaling cascades are those of a channel (fungi) (15) versus those of a G protein-coupled receptor (in animals) (16).

The high variety of photoreceptors is noteworthy, as they could be relevant for the different microbiome members inhabiting animals, including humans, thereby influencing immune system function (outer skin microbiome; Ref. 17) and indirectly physiology and behavior. It is clear that at least microorganisms of the outer skin are exposed to light changes. How these microorganisms are influenced by environmental light is currently unknown. Of note, a bacteriochlorophyll of unknown origin has been suggested to function as a “wavelength converter” and therefore a photosensitizer in the deep-sea fish *Malcosteus niger* (18–20).

**FIGURE 2.**
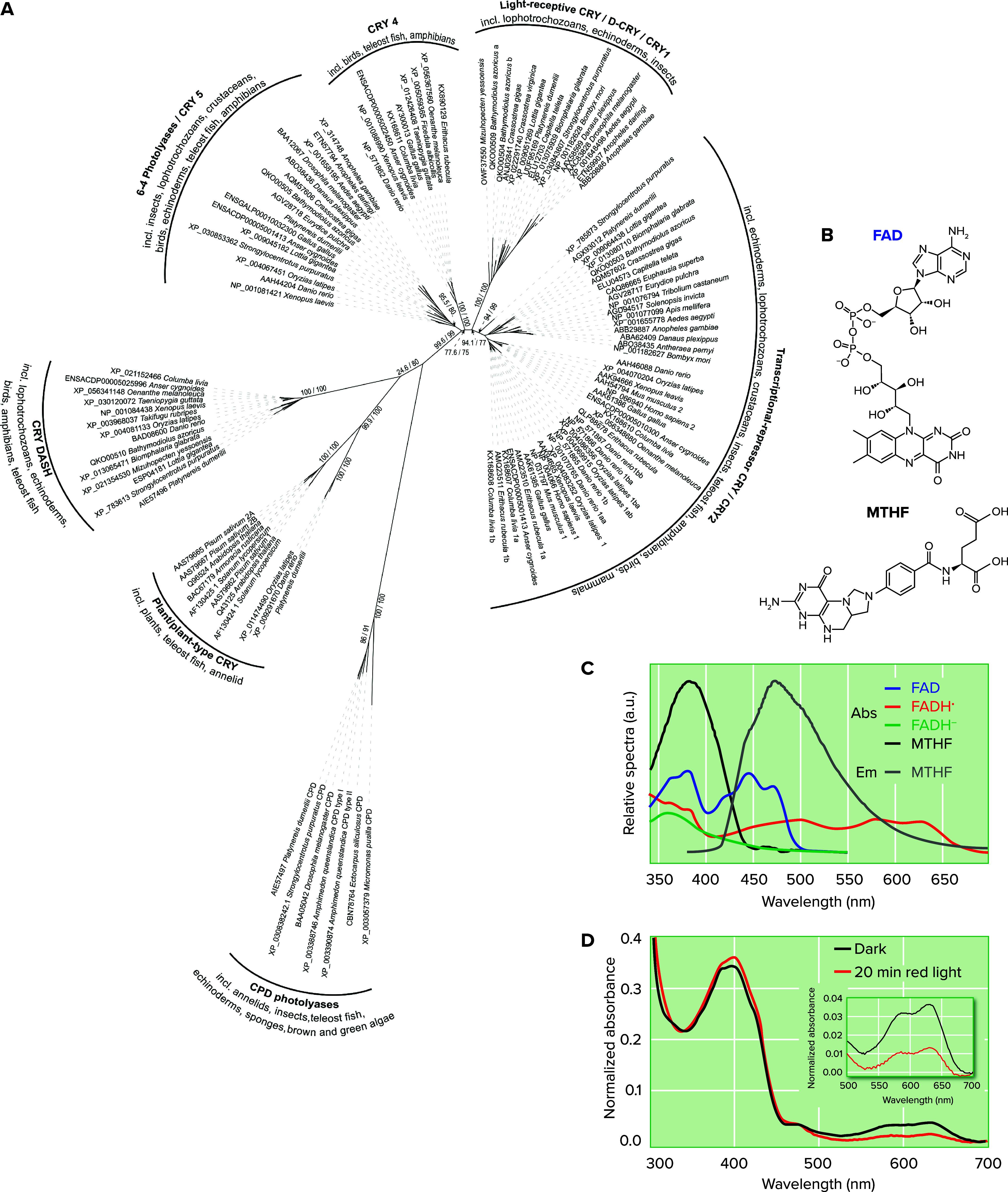
**Cryptochromes/photolyases are a diverse group of animal photoreceptors with at least 6 ancient bilaterian groups***A*: phylogenetic tree of the proteins of the cryptochrome/photolyase families. Branch support was analyzed with IQ-TREE (22) with the ultrafast bootstrap approximation (23) with 10,000 bootstrap alignments plus SH-like approximate likelihood ratio test (24) with 10,000 replicates. Supporting values are presented as SH-aLRT support (%)/ultrafast bootstrap support (%); for readability, only values on major branches are depicted. Numbers represent Genbank or ENSEMBL accession numbers. *B*: chemical formula of the flavine adenine dinucleotide (FAD) and methenyltetrahydrofolate (MTHF) cofactors. *C*: absorbance spectra of different FAD redox states that can occur in cry/phl proteins indicating their photosensitivity and absorbance (Abs)/emission (Em) spectra of MTHF (25). *D*: *Platynereis dumerilii* plant-like cry (pt-cry) can respond to red light. The pt-cry absorbance spectrum was determined in the dark and after illumination with red light for 20 min on ice. The comparison of the resulting normalized spectra shows a strong signature around 400 nm likely arising from MTHF, and an FAD absorbance signature starting from approximately 500 nm up to 700 nm (see *inset*), which decreases in intensity after red light illumination probably due to conversion of the neutral FADH° radical to a fully reduced state of FAD (H. Vu and E. Wolf, unpublished data). CPD, cyclobutane pyrimidine dimer.

This high variety of photoreceptors outside animals might also encourage screening for possible non-opsin, non-cryptochrome light sensors in the various animal genomes that become available. Even within the animal opsins and cryptochromes, it became clear that different subgroups can disappear, such as pt-crys (cryptochromes orthologous to cryptochromes in plants) that were lost in insects and mammals (Ref. 21; FIGURE 2A) or Go opsins that are absent outside marine organisms (26), likely due to the different evolutionary niches inhabited by the organisms. PYP and LOV photosensors exhibit PAS domain structures. These structural domains are found in a variety of animal proteins (often involved in chronobiological processes), whereby the primary amino acid sequences are only relatively little conserved compared to their nonanimal counterparts (14). The notion that PAS domain proteins can function as light receptors in various eukaryote groups leaves the question open if this could also be the case in specific animal phyla. The biochemical verification of any putative new photoreceptor is, however, the labor-intensive, yet critical, experiment to ultimately determine its photosensitivity.

## Animal Opsins and Cryptochromes

Even though “just” two classes of protein-based photoreceptors have been shown to exist so far in animals, their biological functions and ecological implications are far from being fully understood. This is likely due to their complexity:

*1*) Several different opsin and cryptochrome families exist, each with animal group-specific multiplications and loss patterns even in relatively closely related species (21, 26, 27). Animal opsins, seven-transmembrane proteins, which signal via different G-protein signaling cascades, were already present as nine distinct protein subfamilies at the base of all bilaterally symmetric animals, which subsequently further diversified (26, 28). Similarly, cryptochromes, which are closely related to UV-activated DNA repair enzymes called photolyases, form several ancient subgroups (FIGURE 2A). Several of those subgroups display an important functional diversity, including blue-light photoreception, light-dependent DNA repair, transcriptional control, and even combinations of those functions (e.g., Refs. 21, 29, 30).

**FIGURE 3.**
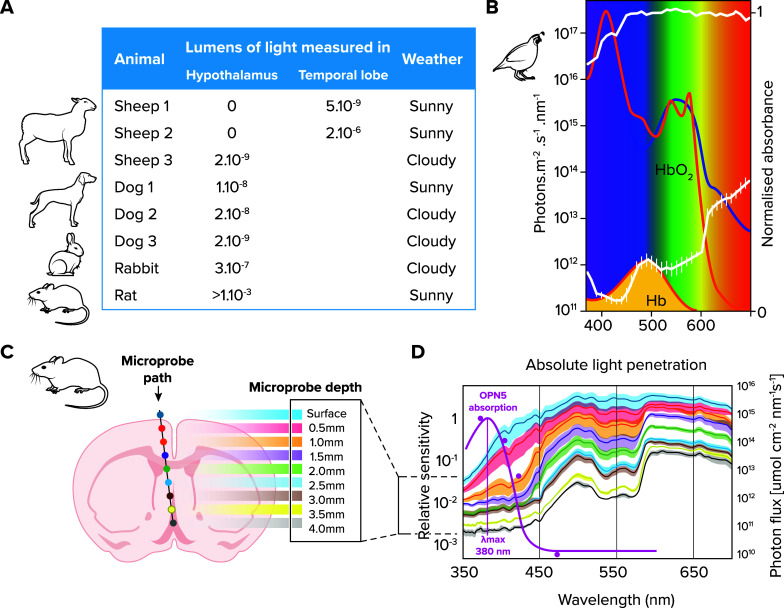
**Examples of amount of light that penetrates animal tissues** *A*: amount of light on a photovoltaic cell inside the respective brain tissue, when the head of the animal is exposed to daylight with weather conditions as indicated. Adapted from Ref. 48, with permission from *Annals of the New York Academy of Sciences*. *B*: amount of light reaching inside the bird brain. Image from Ref. 49 and used with permission from Springer. White top line: daylight; lower white line: light at the level of the hypothalamus; red and blue lines: absorption lines for deoxygenated (red) and oxygenated (blue) bird hemoglobin; and orange line: action spectrum of vertebrate ancient opsin. *C* and *D*: light penetration into the mouse brain. Image from Ref. 50 and used with permission from *Nature*. *C*: location of measurement device in relation to spectra shown in *D*. Purple line: relative sensitivity of the nonvisual photoreceptor opn5.

*2*) Many diverse tissues and cell types express different opsins and cryptochromes. A striking example is presented in a study of zebrafish that reveals that these fish express as many as 42 opsins all over their body (28). For at least 27 of these opsins, action and/or absorbance spectra exist, coupled with additional functional and/or signaling information (28, 31–40). The fish opsins, for which proven light sensitivity exists, have been shown to be expressed in the eye, brain, fin, heart, skin, gills, muscle, pineal, pituitary, and testis (28). Evidence from 20 years ago already established that even the zebrafish heart and kidney cells are directly light sensitive, as are multiple cell lines (41–43). Fish also possess light-sensitive crys (like a CRYII-class member, termed cry1a (44) and by molecular similarity likely others (45), like cry4 and pt-cry (FIGURE 2A). A large number of expressed opsins and additional cryptochromes is likely a general feature of ray-finned fish (Ref. 46; FIGURE 2A). Thus fishes can basically be viewed as “swimming light sensors.” While reduced numbers exist in amniotes (46), individual members, like opn3 in mammals, can be detected in many tissues and cell types (47) and light reaches deep even into various vertebrate tissues, including the brain (FIGURE 3). It is at present unclear, what different expression levels might mean or which levels are or are not relevant. For example, in *Drosophila* the opsin Rh1 has been implicated in temperature sensation of the larval body wall, although its levels are too low to be detected by antibodies or using the GAL4/UAS system (51). Does this imply that even the lowest expression levels have to be functionally considered? Do expression levels impact the sensory properties (photon detection in infrared versus the visible light range) as might be indicated by this study?

*3*) Multiple members are expressed in the same tissue. In the brain, these cells can be interconnected within the same processing pathway, forming what appear to be circuits that are sensitive to light at different steps. This has been pointed out for mammalian Opn3 (47) but can also be observed for *tmt-opsins* in zebra- and medakafish (52–54).

*4*) Until now it is difficult to predict the sensory and signaling properties of opsins and cryptochromes, which means that they need to be experimentally determined. Opsins can function in two different ways: as monostable pigments, they change the isomerization state of the cofactor retinal by light [usually from 11-cis to all-trans on the protein level referred to as rhodopsin (R) into the metarhodopsin (M) conversion,] and the return from the M to R state then requires a separate photoisomerase protein for reconversion of the retinal. Other opsins function as bistable pigments, i.e., after the change of rhodopsin (R) into the metarhodopsin (M) isomerization state by light, light also reconverts the M into the R state. These biochemical differences translate into different responses in the natural environment. While for monostable opsins the amount of signaling is determined by the light intensity of its main activating wavelengths, and the kinetics of the photoisomerase, the signaling of bistable opsins is a direct function of the spectral ratios for the R/M- and M/R-state conversion. One might consider monostable opsins as “light switches” responding once to a specific wavelength range (until reactivated, depending on the availability of the relevant, typical 11-cis, retinal), while bistable opsins provide constant monitoring of the environmental spectral composition. Importantly, even opsins within the same species that share high sequence similarity show different properties for their mono- versus bistability, as exemplified by TMT1 versus TMT2 opsins in medaka (55). Once these properties are known, they can be used to calculate the relative amount of signaling under relevant environmental spectra (3, 142), which is important to understand how any given opsin will actually function in the environment relevant to its organism.

Cryptochromes show similar complex and hard-to-predict biochemical features. Six groups of cryptochromes are likely ancestral to bilaterian animals (Refs. 21, 56; FIGURE 2A). The founding member of type-I Crys was identified in *Drosophila* (57, 58); various members have been found in protostomes and deuterostomes and wherever tested can likely function as photoreceptors (Refs. 59–63 and see below), but type-I Crys disappeared from vertebrates (Ref. 21; FIGURE 2A). Work on the type I *Drosophila* Cry (dCry) located in brain interneurons has shown that this Cry responds to different wavelengths (blue vs. UV/deep violet) by activating different signaling pathways. While blue light ultimately results in transcriptional changes of the core circadian clock (57, 58), UV/deep violet changes membrane conductance by impacting ion channels and by modulating fly behavior (64). The cryptochromes identified in mammals are the founding members of a different class (type II), most of which have so far nonphotoreceptive functions (exception: zebrafish cry1a mentioned above; Ref. 44), and it is thought that their protein structure typically does not permit the stable binding of the critical cofactor FAD (65–67). However, a closely related subgroup (group IV) recently identified in birds/reptiles, amphibians, and fishes contains members that stably bind the FAD chromophore, are light sensitive, and are thought to mediate light-dependent magnetoreception in birds (68, 69). Finally, crys closely related to cryptochromes from plants (pt-crys) are present, yet uncharacterized, in protostome and deuterostome aquatic animals, including teleost fishes (Ref. 21; FIGURE 2A). Our analyses show that they can also function as photoreceptors in animals, given that the pt-cry of the marine bristle worm *Platynereis* binds the cofactor FAD and MTHF and can be photoreduced by light (FIGURE 2, B–D). Like other light-sensitive crys, *Pdu-*pt-cry exhibits a rather broad absorbance range (FIGURE 2D). Of note, the FAD cofactor can adopt different redox states in type I-, type IV-, and pt-Crys: type I and IV Crys bind oxidized flavin (FAD_ox_) in their dark state, which gets photoreduced to the anionic FAD°^-^ radical in type I Crys (e.g., in fruit fly dCry and Pdu L-cry; Refs. 63, 70) and to the neutral FADH° radical in type IV Crys (68). Ecologically this translates into different spectral sensitivity ranges in a given environment, providing examples that the determination of spectral response ranges is difficult to predict from protein sequences but requires biochemical analyses. The animal pt-Cry member from the marine bristle worm appears to bind MTHF and FADH° in its ground state, which is converted into fully reduced FADH_2_ after red light illumination (FIGURE 2, B–D). While FAD_ox_, FAD°^−^, FADH_2_, and MTHF absorb in the blue light and near UVA range, the neutral FADH° radical shows a characteristic absorbance signature in the red light range, i.e., between 550 and 700 nm (FIGURE 2, C AND D). This general broad absorbance range of photosensitive cryptochromes enables them to convey relative spectral intensity changes, which provides organisms with detailed information about their light environment.

All these aspects show the complexity of nonvisual photoreceptors and their differential signaling in animals, which we have only started to understand, especially considering the complexity of their combinatoric (53) and other environmental cues, such as temperature (71).

## Natural Light Is Very Important

The information above allows the conclusion that natural intensity and light spectra are highly influential on animal physiology and behavior, yet natural light is not what model systems are typically exposed to when studied in the laboratory (FIGURE 1, A, B, C, H, AND I vs. J–L). How would the understanding of several neuroscience and physiological paradigms change if the relevant experiments were performed under naturalistic light (cycles)?

### Light as a Critical Factor for Normal Development and Health in Mammals

While the eyes are the light sensory structures humans are most consciously aware of, cells with functional opsins are present in several cell types and tissues outside the visual system even in mammals. In mammals, three main opsin groups have been left from the ancestral vertebrate status: the opn3/ciliary opsin group (including encephalopsin: often referred to as opn3 itself, as well as all short, medium, and long wavelength sensitive opsins present in rods and cones); the opn4 group (with a single mammalian member melanopsin, also referred to as opn4); and the opn5 group (again with a single mammalian member, referred to as opn5) (16, 72). While opsins might not always function as light receptors, even in the presence of retinal (see below), all mammalian members of these groups have been shown to be able to function as photoreceptors in several tissues. Recent reviews provide extensive coverage of 15 different functions so far that are assigned to opsins regulating mammalian physiology in a photon (i.e., light or thermal radiation)-dependent manner by their presence outside the eye in adipose tissue, skin, brain, blood vessels, smooth muscles, and testis (16, 72). Furthermore, opsins present in a subset of mammalian retinal ganglion cells (RGCs) do not only contribute to circadian clock entrainment but also significantly to normal eye development (prevention of myopia), as well as the regulation of mood and motivation (16, 72–74, 143). Finally, even the assumption that the eye’s rods and cones exert purely visual functions is incorrect, as these cells, via their connection to RGCs that entrain the mammalian central circadian clock, also contribute to nonvisual functions. Specifically, they provide additional spectral information for the fine-tuning to dusk and dawn (75). In the same logic, it is thus also likely that those RGCs that mediate light impact on mood and motivation by directly connecting to the perihabenular brain nucleus are also modulated by additional spectral input from rods and cones. These complex interconnections clearly suggest that seemingly simple solutions, like the nocturnal red shift implemented on smartphones and computer screens, might lessen the impact of the artificial light but our light sensory systems will still be perturbed by artificial light. As a consequence, it will be important to educate from early childhood onwards about “light hygiene.” This should include education about regular exposure to natural illumination and how natural spectra and intensity changes (including bright days and dark nights; FIGURE 1) are absolutely critical for normal (eye) development and physiological and mental health by direct action and via their impact on endogenous timing systems (73, 76, 77). Of course, these exposure times need to be adjusted to the individual latitudinal position and genetic background.

### An Example of Natural Light Discrimination: Organisms Have Always Been Living under the Light of Sun and Moon

The human-centric view of a day-active organism often neglects that natural light at terrestrial and water surface areas does not only consist of sunlight but also moonlight. These two light sources provide very different temporal information to the endogenous temporal oscillators of animals. Over decades light used in laboratory settings to entrain daily oscillators has been considered to equal sunlight.

**FIGURE 4.**
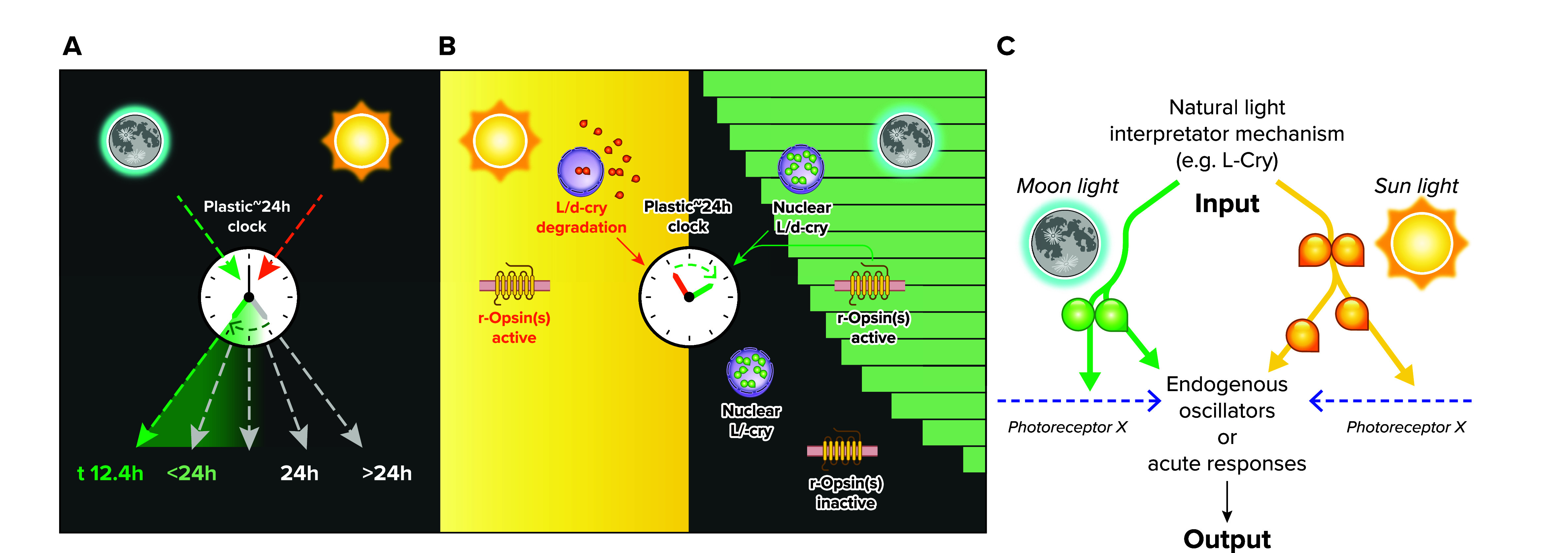
**Sun- and moonlight are relevant light sources to tune animals’ endogenous timers that govern physiology and behavior** *A*: the period length of the daily clock is plastic and depends on the intensity of the environmental light. *B*: the combinatorial signaling of different photoreceptors helps organisms to decode the type of natural light. dCry, *Drosophila melanogaster* type I cryptochrome; L-Cry, *Platynereis dumerilii* type I cryptochrome; r-Opsin(s), rhabdomeric-type osins. *C*: schematic representation of how a light interpretation protein, like L-Cry in *Platynereis dumerilii*, helps an organism to appropriately adjust its different inner timing machineries. Images adapted from data from Refs. 7, 63, and 144, with permission from *Proceedings of the National Academy of Sciences USA*, *Nature Communications*, and *Nature Communications*, respectively.

However, a different view arises from studies of animals that use lunar light to set their endogenous timers (FIGURE 4, A–C). Such moon-controlled timing is particularly well documented in the marine environment and used to synchronize reproductive physiology and behavior within and across individuals of a given species ranging from brown algae to corals, worms, urchins, fishes, and reptiles (78–81). In the marine bristle worm *Platynereis dumerilii*, moonlight is used to set its monthly oscillator and to also tune the period length of its plastic 24-h clock to synchronize the swarming and spawning to the dark hours of the night, just before moon rise (Refs. 7, 63; FIGURE 4, A–C). In both cases, the worms need to discriminate moonlight from sunlight. In the case of the entrainment of the monthly oscillator, the worms are also able to discriminate between moon phases, as the different individuals of a population synchronize to the same moon phase. In essence, the moon phase is encoded by the duration and intensity of the moonlight on the sky. For the decoding of these features, the animals use a type I cryptochrome photoreceptor (L-Cry; FIGURE 2A) expressed in brain and adult eyes that exhibits different biochemical and cell biological properties depending on the type and duration of the light exposure (Refs. 7, 63; FIGURE 4, B AND C, and see below).

It has been known for decades that the period length of the conventional ∼24-h periodic (and hence “circadian”) clock is in fact not fixed but is instead plastic, depending on light intensity (Refs. 82, 83; FIGURE 4A). The physiological relevance of this light-dependent plasticity had remained unclear, however. Experimental evidence from *Platynereis dumerilii* suggests that this plasticity should be viewed from the perspective of natural light, i.e., sun- and moonlight. Under these naturally quite different light intensities, animals can make use of the intrinsic light-dependent period length plasticity to adjust their ∼24-h clock to their relevant ecological niches (FIGURE 4, A AND B). In some organisms, like the nocturnal worm, L-Cry is used to appropriately change the period lengths (7). In diurnal organisms, like the fruit fly *Drosophila*, the ortholog dCry is used to prevent the impact of nocturnal light on its circadian clock, as for these organisms an adjustment to the moon would not be useful (Refs. 7, 63; FIGURE 4, B AND C). Ultimately, it is the integrated information from photoreceptors located in the eye and in the brain that is likely used to discriminate between different types of light for an appropriate setting of the plastic ∼24-h clock (FIGURE 4, B AND C).

It is noteworthy that the plasticity shifts of the endogenous ∼24-h oscillator observed in marine bristle worms in relation to the moon phase also occur in the sleep studies of healthy subjects and bipolar patients (Refs. 84–87 and see discussion in Ref. 7). While for humans it is at present totally unclear what causes these shifts, the comparison to the marine worms shows that such behavioral (and associated physiological changes) according to moon phase can naturally occur and, in the case of the marine worms, likely provide adaptive advantages (7, 63). It might well be possible that humans maintained “evolutionary remnants” of such moon-cycle-dependent changes. In any case, it emphasizes the fact that humans are just another type of animal and as such are strongly interconnected with the environment and biological timers. Being aware of this fact will help to better understand physiological and behavioral changes that can even impact responses to medical treatments (88).

### An Example of the Many Unknown Roles of Nonocular Light Receptors: Light in the Deep Sea

The physiological and psychological health of humans is interconnected with our planet’s health. The oceans are key determinants of planetary health. At the same time, the oceans are the cradle of evolution and hence the environment in which animal sensory and physiological systems originally evolved. The oceans comprise diverse environments, but most of it is deep sea, a vast biosphere typically defined as the habitats below 200 m of seawater. These deep ocean regions designate the waters beyond the photic zone, meaning there is insufficient illumination for net photosynthetic primary production (89). Up to a 1,000-m depth is the twilight zone with residual sunlight that finally diminishes. This residual light ranges in a limited band around 475 nm (Ref. 90; FIGURE 1). Below a 1,000-m depth, sunlight is totally absent (91). Because moonlight is about 10^5^- to 10^6^-fold dimmer than sunlight (63), it will penetrate the water column less deeply than sunlight. However, light and photoreceptors are even relevant in these regions that are not reached by sun- and moonlight: an estimate suggests that 75% of marine organisms exhibit bioluminescence (92). Also, the bioluminescence of deep-sea organisms is centered around 475 nm, while emissions also exist around 410 nm and up to 710 nm (93). Bioluminescent species are found in bacteria (94, 95) and in 13 phyla of invertebrates (92), while the only bioluminescent deep-sea vertebrates known so far are fishes (91). Bioluminescence is used in prey-predator interactions, either as a means of camouflage, attraction, or repulsion. It is also used as a means of communication for reproduction (93). Other sources of light might exist locally. For example, low-level thermal and nonthermal radiations have been measured at deep-sea hydrothermal vents >1,000 m deep (Ref. 6; FIGURE 1G). Perceiving light via photoreceptors is therefore likely a critical sense even for deep-sea organisms. Photons in the deep sea can influence intra- and interspecific interactions (91) and are used as navigational cues (96, 97). However, the exact biological functions are still often unclear. While much of the underlying molecular and cellular mechanisms remain unknown, a few studies provide first insights about the sensitivity and biological relevance of light sensory systems in the probably largest biosphere in volume on earth.

Deep-sea organisms have evolved different strategies that vary with depth to optimize dim light perception. These strategies have mostly been studied in deep-sea fishes, crustaceans, and cephalopods, which actually exhibit adapted functional eyes to improve their efficiency at perceiving light. The adaptions include sharper retinae, dorsal tubular eyes in the mesopelagic zone (200- to 1,000-m depth) or smaller eyes with wide pupils in the bathypelagic (1,000- to 4,000-m depth) and abyssal (<4,000-m depth) zones, reflective layers at the rear of the retina, rod-dominated retina, massive rhabdoms, or pigmented lenses (91, 98). These mechanisms provide not only increased sensitivity but also adapted acuity to a visual landscape that goes from extended scenes to point-sourced emissions as animals dive through the water column (91).

As outlined above, photoreceptors present in the eyes are not only limited to vision. A study analyzing the genomes and transcriptomes of 101 teleost fishes for “visual opsins” reported that while these fishes have lost certain types, there is a significant increase in the number of *rh1* (rod opsin) genes up to 38 in the silver spinyfin (*Diretmus argenteus*, 280- to 2,000-m depth; Refs. 99, 100). At present it is unclear where these many *rh1-*type opsins are expressed. The two zebrafish *rh1-*type opsins are expressed in various tissues outside the eye, including fin, heart, skin, brain, pineal, gills, and testis (28). Hence, the term visual opsins in teleosts is misleading, and these genes have to be equally considered as nonvisual light receptors.

At hydrothermal vents, light emissions show increased intensity at the far-red and near-infrared parts of the spectrum (650–1,050 nm) because of the >350°C temperature of the vent fluid (6). Emissions within 400–700 nm also exist (101). Consistently, evidence for various photopigments that cover spectral ranges for maximal sensitivity from ∼447 nm to ∼670 nm exists but does not go beyond the description of their existence and is typically focussed on structures that by their morphology are considered photoreceptors (102). Given the broad expression of opsins and cryptochromes in nonocular tissues as outlined in the sections above, it is clear that such expression domains need to be tested for. Below are representative examples of the current knowledge that clearly demonstrate that even life under the absence of sun- and moonlight exhibits a rich variety of photoreception.

The deep-sea shrimp *Rimicaris exoculata* is considered endemic to vent ecosystems. It swarms on active hydrothermal vent chimney walls of the Mid-Atlantic Ridge, between 1,500 and 4,200 m deep, close to the ∼350°C fluid, in a gradient ranging from 3 to 32°C (103). They possess nonimage-forming photosensory organs containing enlarged rhabdoms with up to 3,500 photoreceptors (104) that possess a photosensory pigment with a peak sensitivity of 500 nm, likely rhodopsin (97, 105). The Pacific vent crab *Bythograea thermydron* lives on vents at a 2,500-m depth in the Pacific (106). The planktonic larvae (called zoea and megalopa) possess a rhodopsin-like photopigment whose sensitivity shifts from 447 nm in the zoea rhabdoms to 479 nm in the megalopa larvae. While there is no evidence of rhabdoms in adults, they possess still photopigments with a sensitivity shifted toward 489 nm (107). It has been suggested that the evolution of the photopigments sensitivity and an eye-type structure would be beneficial for the larvae that evolve first in the twilight mesopelagic zone and later settle at deeper depths at hydrothermal vents (107). A similar shift in sensitivity through the life cycle has also been reported in several teleost fishes, whose larvae that develop in shallower waters express mostly RH2 cone opsins, while adults that live deeper in the twilight zone express mostly RH1 rod opsins (see above). The RH2 repertoire reaches up to seven genes in the bigfin pearleye *Scopelarchus michaelsarsi* (250- to 500-m depth; Ref. 108).

Deep-sea dragonfishes such as *Aristostomias sp.* and *Malacosteus sp.* emit bioluminescence both in the blue part of the spectrum (479 and 469 nm, respectively), and in the far-red part of it, between 700 and 750 nm (109). The perception of red/far red light in these species is considered useful for intraspecies communication or to illuminate prey, although it should be noted that long wavelength light will get very quickly quenched in water (3, 4). Both fish have pigments shifted toward longer wavelength compared to other deep-sea fish, with peak sensitivities around 515 and 550 nm, respectively (110). One pigment would be a rhodopsin, while the other would be a porphyropsin, an opsin binding a 3,4-dehydroretinal as cofactor (110–112). It has also been shown that *A. tittmanni* (15- to 2,000-m depth; Ref. 113) possesses a third pigment with a peak sensitivity between 586 and 590 nm (111). The hypothesis of another porphyropsin with a theoretical peak sensitivity at 669 nm has been proposed in *A. tittmanni* but not proven (112). *Malacosteus niger* (500- to 900-m depth; Ref. 114) perceives longer wavelength as well but with photopigments that are different than in *A. tittmanni*. There is a peak sensitivity around 670 nm (110) that has been linked to a mixture of defarnesylated and demetallated derivatives of bacteriochlorophylls c and d that would act as photosensitizers for pigments of shorter wavelengths (18, 19).

Various expression studies have revealed further candidates and presence/absence patterns. A comparison between the deep-sea shrimp *Alvinocaris longirostris*, which lives at hydrothermal vents and cold seeps <1,000 m deep, and the shallow-water species *Palaemon carinicauda* indicated that the deep-sea shrimp presents a reduced number of opsins: 5 versus 13, respectively. Phylogeny showed that *A. longirostris* has no short wavelength-sensitive opsin (vs. 3 in *P. carinicauda*), 1 middle wavelength-sensitive opsin (vs. 5), and 4 longwave-sensitive opsins (vs. 5) (115).

Behavioral and transcriptomics data in the hydrothermal vent mussels *Bathymodiolus azoricus* (840- to 3,350-m depth; Ref. 116) showed a clear dominant tidal (12.4-h) signature on the deep seafloor (1,700-m depth). However, while these mussels do not possess any morphologically distinct photoreceptor cells, an artificial 12:12-h light-dark cycle under otherwise constant conditions shifted this dominant tidal pattern to a dominant daily (∼24-h) pattern. This suggested that endemic deep-sea vent mussels can also perceive light (117). Consistently, two type-I putative light-receptive cryptochromes have been reported in *B. azoricus* (117) but remain to be further characterized. In summary, there is light in the deep sea and plenty of light receptors in deep-sea organisms, but so far we have very little knowledge of their functions.

## Functions Other than Light Perception?

The discovery of photosensory proteins outside the visual system has always been accompanied by the debate as to what extent such presence might be connected to light-independent functions. In the context of this review, we consider the sensation of thermal radiation (i.e., temperature) as a type of “light” perception, because thermal irradiation or infrared light also involves the capture of photons. Strong evidence for nonphoton capture-based functions of opsins and cryptochromes is much more scarce than functions connected with photon capture (e.g., examples listed here and in Refs. 16, 72, 118–120). Also, photons reach many places that were previously considered “dark”: it is meanwhile well-documented that light can travel through various tissues, including mammalian and avian brains (48–50, 121, 122), even reaches the retinal layer of unborn mice in the womb (123), and is even present as bioluminescence in the deep sea, possibly in >75% of animals (92) and emitted by hydrothermal vents (Ref. 6; FIGURES 1G AND 3).

Thus it is impossible to conclude based on the environment that any given photoreceptor might not be excited by light or temperature. Furthermore, the absence of an effect is unprovable. There are two examples for opsins and one for cryptochromes for which the accumulated evidence indeed strongly suggests light/radiation independent roles. In *Drosophila melanogaster*, opsins were found in the mechanosensory Johnston organ of the fly antenna and loss-of-function mutations cause lowered mechanosensory functionality (124). This mechanosensory modulation function does not require a retinal-based chromophore (125). This might represent a loss of an originally existing light-depending mechanosensory modulation, as in the bristle worm *Platynereis dumerilii*, an orthologous opsin functions in a light-dependent manner in likely mechanosensory cells to modulate the worms’ undulatory “breathing” movements (126). It is also noteworthy that the cellular signaling of these kinds of rhabdomeric opsins typically involves membrane stretch, which could provide a possible mechanistic explanation for its role in the bristle worm (126). Another light-independent function has been suggested for mammalian opn3. While several opsins expressed in skin, including opn3, perform light-dependent functions, such as the promotion of wound healing (127), opn3 modulates melanin production in human melanocytes in a light-independent fashion by impacting melanocortin 1 receptor signaling (128). Finally, mammalian cry II-type cryptochromes function as transcriptional repressors in the core circadian clock and have lost their direct light sensitivity (Ref. 129 and above). The binding pocket of its typical cofactor FAD is modified to competitively bind the COOH terminus of the SCF^FBXL3^ ubiquitin ligase, ultimately controlling Cry’s ubiquitinylation and degradation (67). However, as FAD can still bind the discussion about complete light insensitivity may remain open. In summary, even at “unconventional” sites, opsins and cryptochromes typically sense light and may use this property for the modulation of cellular processes to likely optimize them to naturally occurring changes. As their primary functions are often modulatory, it is more difficult to determine their roles with and without light.

An interesting case is magnetoreception, which likely utilizes quantum physical effects enabled by the light receptive function for the detection of another environmental variable, the magnetic field. Specifically, one proposed mechanism suggests that the spin dynamics of a radical-pair system generated by a light-dependent electron transfer reaction is impacted by the magnetic field and thus allows its detection (130, 131). Although many details are still unclear, evidence from different laboratories supports cryptochromes as a magnetic sensor in the fruit fly *Drosophila* and the monarch butterfly *Danaus plexipus* (CRY1 or light-sensitive CRY) (132–135) and birds (CRY4) (68, 69, 136), although this has recently been debated for *Drosophila*. One publication casts principle doubts (137), while another suggests that high levels of free FAD are sufficient to mediate blue light-dependent magnetic sensitivity, even in the absence of cryptochrome (10, 11).

## Outlook: Quantum Physical Effects of Light in Biology?

The biological mechanisms that explain how photons trigger cellular (and subsequently organismal) responses currently focus mostly on biochemical signaling cascades. However, as outlined above, processes that involve photons, electrons, protons, excitations, chemical bonds, and electronic charges are by definition quantum effects, and an understanding of their dynamics requires quantum mechanics (138, 139).

Much has been debated about whether the human eye might be sensitive to single photons (140), and quantum physic-based experimental designs have been employed to test this (141). However, the biological relevance of such a possible high sensitivity is unclear given that humans are a diurnal (i.e., day active) species and our vision matches our activity patterns, which occur when there is relatively bright light. Thus it is unlikely that there is evolutionary pressure to select for the most efficient light detection mechanisms in the human eye. On the other hand, as outlined in different examples above, photoreceptors exist in various internal tissues (e.g., mammalian brain) and places (deep sea) that would benefit from the selection of optimized high quantum efficiency. One specific example could be a light-sensitive cryptochrome in the bristle worm *Platynereis* that is highly light sensitive to detect moonlight but also discriminates between sun- and moonlight (see above). This could be made possible due to different quantum efficiencies in the FAD photoreduction reactions in its dimer, one reaction being exquisitely quantum efficient for moonlight detection, while the second reaction is much less efficient to ensure that sunlight, but not moonlight is sensed (63). An understanding of how high quantum efficiencies are achieved and mechanistically optimized on the molecular level would certainly benefit from interactions between quantum physics and molecular photobiology: further improving the comprehension of the many impacts of light on physiological processes.

## Clinical Relevance

Light has many more effects on mammalian physiology and behavior than just vision. Experiments on vertebrates and invertebrates have been crucial to start unraveling these functions. Light can directly affect biochemical processes, such as the well known production of vitamin D, but also the production of other substances, such as urocanic acid (UCA). UCA can cross the blood-brain barrier in mammals and affects learning and memory. Other non-visual functions are exerted via three currently known non-visual opsin photoreceptors. These are critical for human circadian and sleep-wake cycles, the direct regulation of mood and motivation, the prevention of myopia during eye development, and at least 13 other documented functions in mammals. It should thus be clear that exposure to natural light spectra and intensities is a highly relevant health factor. Education about the right light at the right time is important for medical professionals, teachers, and also the general public. Teaching only about the carcinogenic aspects of sunlight is clearly too simplistic. “The dose makes the poison” also applies to light.

For the biologically interested reader, this review also covers several aspects of the importance of light to other animal systems (including lunar cycles and the deep sea) to give a flavor of how little is currently understood about the effects of light, the distribution of photoreceptors and their role in human and planetary health.
